# Early Detection of Undiagnosed Hypertension Based on Occupational Screening in the Hotel and Restaurant Industry

**DOI:** 10.1155/2018/6820160

**Published:** 2018-04-08

**Authors:** Reingard Seibt, Bettina Hunger, Lisa Stieler, Regina Stoll, Steffi Kreuzfeld

**Affiliations:** ^1^Institute for Preventive Medicine of the Rostock University Medical Center, St.-Georg-Str. 108, 18055 Rostock, Germany; ^2^Center for Life Science Automation (CELISCA), Rostock University, F.-Barnewitz-Str. 8, 18119 Rostock, Germany; ^3^Government Safety Organisation Foods and Restaurants, German Social Accident Insurance Institution for the Foodstuffs and Catering Industry, Office of Coordination Potsdam, Potsdam, Germany

## Abstract

Blood pressure is the most important, modifiable risk factor for cardiovascular diseases. Lifestyle factors and also workload are the main, potential risk factors for the development of hypertension. This study focused on the early detection of unknown hypertension by screening employees in the hotel and restaurant industry (HRI). 148 HRI employees without hypertension (mean age: 34 years, men: 45%) self-measured their blood pressure during rest and for 24 hours of a normal workday. Individuals with a resting blood pressure ≥ 135/85 mmHg were classified as hypertensive. A further analysis investigated whether the currently applicable thresholds for hypertension during work, leisure, and sleep were exceeded on a working day. At rest, 36% of the study participants suffered from hypertension, which increased to 70% under workload and 46% during leisure time and dropped to 8% during sleep. Normal nocturnal dipping (10–20%) occurred only in 18% of cases; 78% were extreme dippers (>20%). Occupational hypertension screening is a suitable component of preventive healthcare. Resting blood pressure measurement alone is insufficient for the early detection of risk individuals and should be supplemented by 24-hour ambulatory blood pressure monitoring under working conditions. The impact of workload on blood pressure needs to be given more attention in the guidelines.

## 1. Introduction

Hypertension presents the most significant health risk worldwide [1]. Its prevalence in the population is high and increases with age. In 2015, every fourth man and every fifth woman worldwide was affected by it [2]. A not inconsiderable number of these people do not know that they suffer from it. Since hypertension is also the most important, modifiable risk factor for cardiovascular diseases, its early detection is of great significance.

In addition to genetic, social, and lifestyle-related factors [3, 4], work-related requirements also play a role in the development of hypertension, for example, long working hours [5], shift work [6], and psychosocial factors [7, 8]. It is therefore important to monitor hypertension under the influence of real working conditions.

Office blood pressure measured by physician results in increased blood pressure values in approximately 20% of the general population (white-coat effect), something which does not occur however in self-monitored home blood pressure measurement (home BPM) or in an ambulatory 24-hour blood pressure monitoring (a 24-hour ABPM) [9, 10]. Conversely, masked hypertension is defined as a normal office blood pressure accompanied by an elevated blood pressure on home or ambulatory monitoring [11]. The prevalence of masked hypertension is given as 10–23% in various studies, is associated with male sex, body mass index, and current smoking status [12], and is higher in people with stressful occupations [13].

Thus, office blood pressure is only partially suitable as a diagnostic procedure. For reliable blood pressure measurements, international consensus recommends measuring out-of-office blood pressure such as home BPM or a 24-hour ABPM under normal day-to-day conditions [14, 15].

In comparison to office blood pressure, home BPM and a 24-hour ABPM are also better predictors of hypertensive organ damage and cardiovascular risk [16–19]. To identify higher risk patients early, the use of home BPM and a 24-hour ABPM is therefore indispensable.

In the present study, the blood pressure (BP) of employees in the hotel and restaurant industry was monitored under resting conditions at home and under work and recovery conditions. This branch is known for shift work, long working hours, and work under time pressure [20].

Within the framework of an occupational screening, home BPM was used as a diagnostic procedure to identify undiagnosed hypertension. The subsequent 24-hour ABPM followed with the question of whether a classification into normotensive and hypertensive persons based on the home BP changes under work and recovery conditions. Then, an analysis was carried out to determine whether work and recovery are predictors of hypertension.

## 2. Material and Methods

### 2.1. Sample

The participants in the study were 160 employees in the hotel and restaurant industry (HRI). They came from 11 hotels of the upmarket class in Berlin and the surrounding area. The data were collected within an occupational screening programme from October 2014 to February 2016. The participants took part voluntarily (participation rate: 75%).

Persons with known hypertension and persons already being treated with medication (*n* = 12) were excluded from the statistical analysis; that is, 148 restaurant and hotel employees were included in the analyses (45% men). Among the participants were 49% with standard secondary school education, 45% with high school level, and only one employee with lower secondary school education. The participants were between 18 and 63 years old (Ø 34 ± 9 years); 49% worked in shifts (change between morning and afternoon shift with a small percentage of night shift work (♂: 53%; ♀: 47%)) and 51% in day work (♂: 38%; ♀: 62%).

On average, the employees worked 42 ± 8 hours per week, whereby men worked four hours longer than women (♂: 44 ± 9 hours; ♀: 40 ± 6 hours; *p* = .003). The median employment duration was seven years.

The professional spectrum was diverse: kitchen staff (19%), restaurant and hotel specialists (16%), administration and management staff (33%) as well as room service and housekeeping (9%), reception staff (13%), and other service personnel. The detailed description of the sample is shown in Table 1.

### 2.2. Instruments

The occupational health screening programme with its focus on hypertension diagnosis consisted of three parts: a questionnaire, a self-monitored home BPM, and a 24-hour ABPM.

#### 2.2.1. Questionnaire

A modified version of the shift worker questionnaire for employees of the hotel and restaurant industry [21] was used to collect the sociodemographic (sex, age, size, weight, and education) and work-related data (occupation, shift group, and weekly working hours) and to indicate lifestyle factors (sport, smoker status, and alcohol consumption).

#### 2.2.2. Home BPM

The home BPM served to record resting BP and to determine hypertension [14]. It took place on four days and was carried out using a BOSO medicus BP measuring device (Bosch + Sohn GmbH, Jungingen, Germany) on the left upper arm. The size of the cuff was selected relative to the diameter of the participant's arm [22]. The participants were asked to perform six measurements daily between 06:00 hrs and 22:00 hrs, at intervals from two to three hours, while sitting after a three-minute rest period. The resulting mean values of systolic (SBP) and diastolic (DBP) blood pressure from a total of 24 measurements were evaluated in accordance with the current guidelines of the European Society of Hypertension [14]. Arterial hypertension was diagnosed if SBP was on average ≥ 135 mmHg or DBP ≥ 85 mmHg.

#### 2.2.3. 24-Hour ABPM

The 24-hour ABPM ensued under work and recovery conditions. It was performed using the fully automatic measuring device TM-2430 (Bosch + Sohn, GmbH, Jungingen, Germany). Three cuff sizes were used for the measurements, which were selected in accordance with the size of each participant's upper arm [22]. The measurements were taken on the left upper arm and at 15 minutes intervals during the day (06:00–22:00 hrs) and at 30 minutes intervals during the night (22:30–05:30 hrs). This resulted in a total of 80 individual measurements, which were allocated to the time periods WORK, LEISURE, and SLEEP defined [23] as follows:

- WORK: time between beginning and end of work with recovery periods [24]
- LEISURE: time between end of work and going to bed (including sleep periods on day between end of work and before going to bed)
- AWAKE: time after getting up until going to bed (=WORK + LEISURE)
- SLEEP: time between going to bed and getting up the following morning.

Parallel to these 24-hour ABPM, the participants completed a diary at intervals of 30 minutes. In this, they noted their activities and body positions. This enabled the retrospective allocation of the BP values to the activities and the start of the time periods, as well as the subsequent estimation of workloads.

The mean values and standard deviations (SD) were then calculated for SBP and DBP, and the differences between the BP levels in the three time periods (WORK to LEISURE, WORK to SLEEP, and LEISURE to SLEEP) were determined. The mean BP values of each time period and the differences of the values were used for the statistical analyses.

According to the guidelines of the European Society of Hypertension [14], the hypertension thresholds for the 24-hour ABPM were set as follows: AWAKE ≥ 135/85 mmHg, SLEEP ≥ 120/70 mmHg, and the 24-hour TOTAL BP ≥ 130/80 mmHg. For the WORK and LEISURE periods, hypertension limit values were ≥135/85 mmHg too. Arterial hypertension was diagnosed as soon as the threshold of SBP or DBP was reached.

### 2.3. Data Analyses

The statistical data analysis was performed using the “Statistical Package for Social Science” programme (SPSS 23.0) for Windows (SPSS INC., Chicago, IL, USA). The error probability value of *p* < .05 was considered statistically significant and supplemented by effect sizes. The interpretation of the effect sizes is based on the Cohen conventions [25]. The comparisons of the mean values were carried out by analysis of variance considering several confounder factors (gender and shift work). The Chi^2^-Test was used to test the difference in categorical variables. Correlations between the measurements of home BPM and 24-hour ABPM were analysed with partial correlations (control variables: sex and body mass index). In order to determine the effect of the independent variables on the hypertension diagnosis, covariance analyses were carried out controlled by sex and body mass index. To determine the predictors of undiagnosed hypertension, a binary logistic regression model was calculated in which lifestyle factors and control variables were taken into account in addition to the time periods of the 24-hour ABPM.

#### 2.3.1. Research Ethics

This study was conducted in conformance with the guidelines of the World Medical Association (WMA) Declaration of Helsinki and the ethical principles of medical research involving human subjects amended by the 9th WMA General Assembly, Soul, Republic of Korea, October 2008. The study was approved by the Ethics Committee of the Technische Universität Dresden (EK 250397) and written informed consent was obtained from all participants included in this study.

## 3. Results

### 3.1. Blood Pressure under Resting Conditions (Home BPM)

In the self-monitored home BPM, approximately one-third (36%) of the 148 participants showed elevated BP (mean values SBP ≥ 135 mmHg or DBP ≥ 85 mmHg [14]). These employees were unaware of their hypertension. The average for the resting BP of the whole sample was 128/80 mmHg. The standard deviations (SBP: ±14 mmHg; DBP: ±9 mmHg) however clearly show that the diagnosis of hypertension was determined either by elevated SBP (10%) or DBP (8%), or an increase in both blood pressure values (18%).

Subsequently, participants were classified as normotensives and hypertensives and were studied comparatively (Table 1). For the normotensive participants (64%), the results show an average BP value of 120/75 mmHg (SD: ±9/6 mmHg), for hypertensives 142/88 mmHg (SD: ±10/7 mmHg) (*p* < .001). Other significant differences were found only for sex (*p* = .001) and professional spectrum (*p* = .041).

Approximately half of the participants were employed in shift work (*p* = .194). The professional spectrum was distributed significantly differently between both groups (*p* = .041, small effect). Employees with primarily a physical workload (kitchen and room service staff) showed more frequent hypertension (48%) than employees with primarily mental workload (23%) or “mixed” workload (29%). Within the workload groups, it became clear that almost one-third of the employees in administration and management fields (30%) and almost one-quarter of the kitchen staff (24%) had untreated hypertension (*p* = .099).

### 3.2. Blood Pressure under Working and Recovery Conditions (24-Hour ABPM)

**Figure 1:**
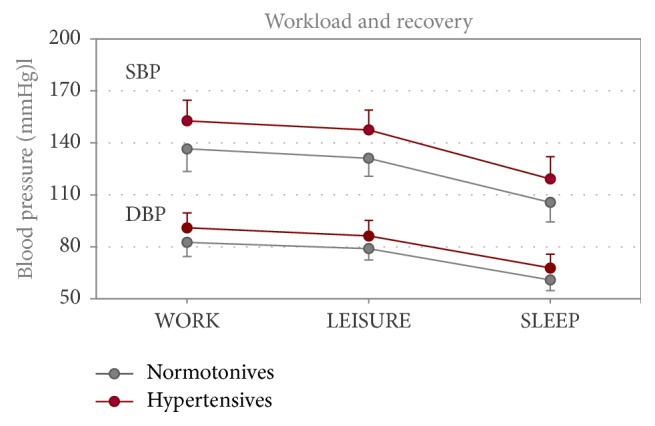
Blood pressure of normotensives (*n* = 95) and hypertensives (*n* = 53) in the time periods WORK, LEISURE, and SLEEP (24-hour ABPM) on a working day.* Note*. SBP: systolic blood pressure; DBP: diastolic blood pressure; means and standard deviations; ANOVA test statistics; significance threshold (two-tailed): *p* < .001; mean blood pressure of the normotensives and hypertensives differed during all time periods; controlled by sex and body mass index.

Due to the influence of sex and body mass index, the expected BP differences between normotensive and hypertensive participants were confirmed for all time periods of the 24-hour ABPM (*p* < .001; *η*^2^ = .140 to .251, large effect) (Figure 1). On average, the normotensive participants' SBP values were 16 mmHg and the DBP values were 8 mmHg lower than the BP values of the hypertensive participants. In the hypertensive employees, all BP mean values of the 24-hour ABPM were found to be in the hypertensive range (*Ø* BP WORK: 153/91 mmHg; *Ø* BP LEISURE: 148/86 mmHg) apart from the SLEEP period (*Ø* BP: 119/68 mmHg). Most noticeable however is the presence of hypertensive mean values in normotensive participants in the WORK period (*Ø* BP WORK: 137/83 mmHg; SD: ±13/8 mmHg). For sex, the results showed no significant effect on BP during the working day. The BMI only had an influence on SBP (*p* < .001; *η*^2^ = .10–.11).

**Figure 2:**
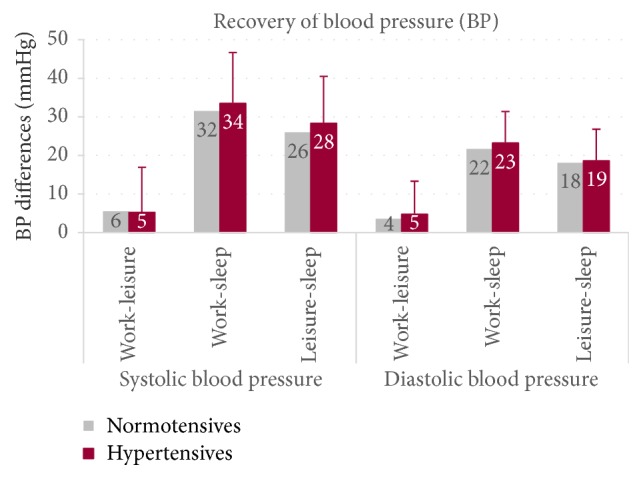
Blood pressure differences between the time periods of normotensives (*n* = 95) and hypertensives (*n* = 53) of the 24-hour ABPM on a working day.* Note*. Means and standard deviations; significance threshold (two-tailed): no significant differences in recovery periods were found for blood pressure of normotensives and hypertensives controlled by sex and body mass index.

The normotensive and hypertensive participants did not differ in cardiovascular recovery after WORK (LEISURE: SBP: *p* = .371; DBP: *p* = .230; SLEEP: SBP: *p* = .312; DBP: *p* = .281) (Figure 2). The drop in BP with an average of 5 mmHg (SBP) respectively 4 mmHg (DBP) was the lowest from WORK to LEISURE, and the most significant between WORK and SLEEP, with an average of 32 mmHg (SBP) respectively 22 mmHg (DBP).

Contrary to our expectations, for hypertensives, the results show a trend towards a more favourable cardiovascular recovery (largest BP difference between WORK and LEISURE). Sex (*p* = .920) and body mass index (*p* = .070) have no influence on the recovery processes. The standard deviations for the BP recovery values however indicate that there were very different physical activities in the WORK and LEISURE periods, respectively, very different individual recreational activities.

In accordance with the recommendations of Middeke [26], the average fall in nocturnal BP can be considered as normal in only 18% of the employees. Moreover, the results show a nocturnal decline in SBP by 29 mmHg and in DBP by 20 mmHg compared to the daytime mean. In most of the employees (78%), these night-time BP values dropped by more than 20% (extreme dipping). Only a few cases were nondippers (3%), respectively, and inverted dippers (1%).

### 3.3. Changes of Hypertension Diagnosis (Home BPM) under Work and Recovery Conditions (24-Hour ABPM)

To detect changes in the BP classification under work and recovery conditions, the BP values for the time periods of the 24-hour ABPM were evaluated using the hypertension thresholds of the European Society of Hypertension [14].

**Figure 3:**
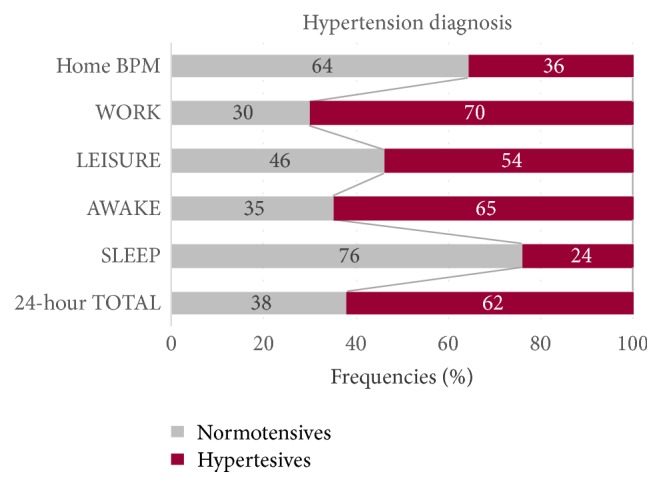
Comparison of hypertension diagnosis by home blood pressure measurement (home BPM) with the time periods of 24-hour blood pressure monitoring (24-hour ABPM) (*n* = 148).* Note*. Criteria of hypertension [14]: home BPM, WORK, LEISURE, AWAKE: ≥135/85 mmHg, SLEEP: ≥120/70 mmHg, and 24-hour TOTAL: ≥130/80 mmHg.

Only around 30% of the employees showed normal BP during WORK. In the LEISURE period, this was true for almost half (46%) of them. For the AWAKE and for the 24-hour TOTAL period, one-third of the employees had normal values (Figure 3).

It is important to note that even half of normotensive employees had hypertensive BP during WORK (56%) and more than one-third had it in the LEISURE period (Table 2). A small number of them (8%) can be classified as hypertensive even in the SLEEP phase. The normotensives had high-normal BP values (SBP: 125–134 mmHg and DBP: 80–84 mmHg) in home BPM. In contrast, a small number of the hypertensives showed normal BP under WORK and LEISURE conditions (6%, and 11%, resp.). These hypertensives only slightly exceeded the hypertension limit (SBP: 135–137 mmHg or DBD: 85–88 mmHg). Around half of all hypertensives (49%) recovered sufficiently during the SLEEP period and showed normal BP values.

Based on the hypertension criteria of the European Society of Hypertension [14], the hypertensives appear to have been more often diagnosed correctly compared to the normotensives with the exception of the SLEEP period. For SLEEP, the opposite is the case: three-quarters of the normotensives, and only one-quarter of the hypertensives were correctly classified by their original diagnosis from home BPM.

There were only slight to medium correlations between the values of home BP and 24-hour ABPM for both the SBP (NT: *r* = .36–.52; HT: *r* = .27–.50) and the DBP (NT: *r* = .27–.46; HT: *r* = .39–.59) in both groups; this means that only a maximum of 35% of the variance between both BP values was declared.

### 3.4. Predictors for Hypertension by Home BPM

Binary logistic regression analyses (method: inclusion) were performed to determine whether the work and recovery periods are predictors which can explain the unknown diagnosis of hypertension and how it is effected by health-related behaviour (sport, smoking, alcohol consume, and body mass index). In addition, sociodemographic variables (sex, age, and shift work) were included in the analyses. Whether there are predictors among these variables has not been found in the literature reviewed here to date.

Moreover, the sociodemographic variables, the variables of health-related behaviour, and the diagnoses for the periods of the 24-hour ABPM were individually subjected to the binary logistic regression analysis. In the second analysis step, a whole model was created which contained all the variables significant in the first step (Table 3).

In the first step, a significant influence on hypertension diagnosis by home BPM was found only for sex and body mass index; this explains 23% of the variance. The best result was achieved simultaneously including the interpreted BP values from WORK, LEISURE, and SLEEP, which allowed explaining 44% of the variance in hypertension diagnosis.

In the whole model, it was shown that only sex and the diagnoses in the time periods LEISURE and SLEEP had a significant influence on the diagnosis by home BPM and could explain a total of 52% of the variance.

Using this predictor model, 82% of the employees from the sample were classified with the correct diagnosis. 83% of the normotensives were predicted correctly; however, 17% were classified as hypertensive. 79% of the hypertensives were predicted correctly and 21% as normotensive (false negative diagnosis).

Men had a 3.6-fold higher risk of hypertension than women. As opposed to normotensives, hypertensives showed a 6.3-fold higher risk of hypertension in LEISURE period and a 5.3-fold higher risk in SLEEP period.

## 4. Discussion

Elevated blood pressure values in the workplace and their health significance have so far been underestimated. Correspondingly, a screening programme was used to diagnose hypertension in employees in the restaurant and hotel industry. The blood pressure status of these employees was analysed under resting conditions (home BPM) as well as working and recovery conditions (24-hour ABPM).

In the self-monitored home BPM, an unexpectedly high number of the participants (36%) were identified as unknown hypertensives (SBP ≥ 135 mmHg or DBP ≥ 85 mmHg). This diagnosis was influenced by gender (*p* = .001) and workload (*p* = .041). Accordingly, the employees with primarily physical workload showed more frequent hypertensive blood pressure values (48%) than those with primarily mental (23%) or mixed workload (29%).

In the 24-hour ABPM, the expected differences in the blood pressure levels between normotensive and hypertensive subjects were confirmed. The BP changes in the 24-hour ABPM reflected normal diurnal variations in both groups at different levels.

It should be emphasised that under working conditions the mean values of systolic blood pressure also in normotensives were in the hypertensive range (*Ø* BP: 137/83 mmHg). During the recovery period after work, the BP behaviour did not differ significantly between both groups. During the LEISURE period, BP dropped only from 3 to 6 mmHg on average (Figure 2), which indicates that a high level of activity was maintained in this period. The standard deviations show clearly that a large variety of leisure activities were carried out.

The recovery effect can only be reliably estimated using the SLEEP period, which guaranteed restful conditions. A normal nocturnal fall in BP by 10–20% (normal dipping) occurred in only 18% of the participants, whereby in 78% of them the nocturnal decline in BP was more than 20%. This phenomenon is called “extreme dipping” and is linked to a higher risk of cardiovascular diseases in patients with untreated hypertension [27]. Independent of dipping, night-time blood pressure values >120/70 mmHg are associated with an increased risk of organ damage [28] and of cardiovascular mortality [29].

In comparison with the home BPM (36% hypertensives), the number of hypertensives rose to 70% under working conditions, to 54% during LEISURE time, and to 62% based on the TOTAL 24-hour period.

Over half (56%) of those who had normal BP values in home BPM were classified as hypertensive under working conditions. At LEISURE, this accounted for 35% and even during the SLEEP period it was still 8%. Contrarily, normal BP was measured in only 6% and 11%, respectively, of the hypertensives under working and leisure conditions, but in the SLEEP period, it accounted for half of the hypertensives. This indicates that only measuring resting BP is insufficient to detect all vulnerable persons.

Pieper et al. [30] had already shown in the 1990s that also for normotensive persons the mean 24-hour TOTAL BP on workdays is higher than on work-free days and remains elevated after work has finished (carry-over effect). In the years following, some studies provided indications of the influence of working conditions on the development of hypertension such as, for example, physical work and incontrollable stress [31], constant demand for attention [32], long working hours [5], job strain [8], and shift work [33]. Over decades, a growing number of studies have investigated the adverse effect of psychosocial workplace factors on hypertension. Overall, the scientific findings of a number of studies have remained inconsistent until today [7]. This has been mainly due to methodologies. There have been a lack of prospective studies on the one hand and a lack of investigations in which 24-hour ABPM has been used under working conditions on the other. Most studies thus far have used office BP to prove correlations between working conditions and hypertension. To date, however, it has not been sufficiently investigated how the blood pressure changes under working conditions.

In relation to maintain current BP thresholds, even under working conditions, there is also still uncertainty in the international guidelines. The fact that the same threshold for BP under resting conditions is used as for average daytime BP in the 24-hour ABMP should be reviewed in future studies.

This study also analysed the participants' lifestyle as a risk factor in hypertension development. An influence of health-related behaviour on BP could not be demonstrated although there were significantly more overweight (38%) and obese employees (24%) among the hypertensives than among the normotensives (28% and 5%, reps.). The number of smokers (41%) and physically active persons (43%) was the same for both groups.

In a separate analysis of health-related behaviour variables in combination with the sociodemographic data, body mass index emerged in addition to sex as a predictor of hypertension by home BPM (variance explanation: 23%). This indicates that high BP was present primarily in overweight and obese men. This correlation is known from epidemiological studies. The men in this sample showed a 3.6-fold higher risk of hypertension than the women. Moreover, the risk of the overweight and obese employees, respectively, was 3.6 times higher compared to those of normal weight. Critical for a reliable diagnosis was the BP level, in addition to sex, in the LEISURE and SLEEP period. 52% of the resulting variance could be explained by the predictor model and 82% of all cases were correctly diagnosed.

It was assumed that BP during work in particular is suitable to correctly predict the diagnosis. The accuracy of the prediction was however influenced by the fact that more than half of those analysed (56%), who measured normal BP during home BPM, showed hypertension under workload. The results of the regression analyses support the assumption that the internationally accepted daytime average for 24-hour ABMP “might be regarded as too conservative by some” [14].

From a methodological perspective, the rest blood pressure values from the home BPM indicate high objectivity and validity. They are based on a total of 24 measurement values and the data collection is in line with the guidelines of the European Society of Hypertension [14]. The strength of the study lies in the analysis of 24-hour blood pressure behaviour under real working conditions, during LEISURE and SLEEP and its comparison to self-monitored home BP. This enabled the study to provide important information on the bandwidth of the physiological responses in a relatively young sample.

A limitation of the study was the cross-sectional design, which was only able to describe correlations but unable to prove causality. The results can however be used to generate hypotheses in future studies on the tendency of correlations and differences.

The generalisability of the study was limited by the convenience sampling of 148 participants; that is, selection effects cannot be excluded. The limitation of representativeness may have been caused by the fact that this sample was a regional one (Berlin region, Germany). Nonetheless, the sample was representative of all German staff in the hotel and restaurant industry with regard to age and sex.

Regardless of these limitations, an occupational screening programme can make an important contribution to preventive healthcare. Various studies have shown that particularly young, male employees with subjectively good health and seldom contact to a physician are not often diagnosed as hypertensive and thus remain untreated [34, 35]. Young persons rarely take part in preventive health checks provided by the health insurance companies [35]. In this study, directly approaching the company doctor made it possible to achieve a high participation rate of 75%. The acceptance of blood pressure measurements was good, even under working conditions.

## 5. Conclusion

The present data indicate occupational screening with home BPM to be an alternative path to detect undiagnosed hypertensive persons. Home BPM should however be complemented by a 24-hour ABPM as a number of presumed normotensive individuals do develop hypertension under daily workload. It is thus crucial to give a critical account of current guidelines which recommend the same blood pressure thresholds for both home BP at rest and the average daytime BP under daily conditions.

## Figures and Tables

**Table 1:** Characteristics of the sample.

	Normotensives	Hypertensives	Group differences		
	(*n* = 95)^1^	(*n* = 53)^2^	Test statistics	*p* values	Effect-size
Age [years; M ± SD]	33.0 ± 8.7	35.2 ± 10.0	*F* = 1.8	.176	-
Sex [%]					
(i) *Male*	35	64	*χ* ^2^ = 11.9	.001	*V* = .293
(ii) *Female*	65	36			
Shift [%]					
(i) *Day work*	53	42	*χ* ^2^ = 1.7	.194	*V* = .107
(ii) *Shift work*	47	58			
Working hours per week					
[Hours; M ± SD]	41.3 ± 7.2	43.6 ± 8.0	*F* = 3.2	.077	-
Health behaviour					
Body mass index (BMI)					
BMI [kg/m^2^, M ± SD]	23.9 ± 3.1	26.9 ± 5.3	*F* = 19.4	<.001	*η* ^2^ = .117
(i) *Normal weight *[%]	65	38	*χ* ^2^ = 16.5	.001	*V* = .332
(ii) O*verweight *[%]	28	38			
(iii) *Obesity *[%]	5	24			
Sport [%]					
(i) *Not at all*	32	40	*χ* ^2^ = 1.9	.393	*V* = .123
(ii) *Occasionally*	21	25			
(iii) *Regularly*	47	36			
Smoking [%]					
(i) *Smoker*	35	51	*χ* ^2^ = 3.7	.054	*V* = .158
Alcohol consumption [%]					
(i) *Not at all*	17	13	*χ* ^2^ = 3.0	.224	*V* = .123
(ii) *Occasionally*	62	53			
(iii) *Regularly*	21	34			

*Note*. *n*: sample; M  ± SD: mean ± standard deviation; [%]: frequencies in %, *χ*^2^: test statistics; *F*: ANOVA; significance thresholds (two-tailed): *p* < .001, *p* < .01, *p* < .05; *η*^2^: eta-square; *V*: Cramer's *V* (correlations 0.1–0.3 weak, 0.4–0.5 medium, >0.5 strong); ^1^Normotensives (SBP < 135 and DBP < 85 mmHg) and ^2^hypertensives (SBP ≥ 135 or DBP ≥ 85 mmHg) were differentiated by their outcomes of home BPM.

**Table 2:** Changes in hypertension diagnosis by self-monitored home blood pressure measurement (home BPM) compared to 24-hour blood pressure monitoring (24-hour ABPM).

24-hour ABPM		Home BPM		Group differences		
	Sample (*n* = 148)	Normotensives (*n* = 95)^1^	Hypertensives (*n* = 53)^2^	Test statistics	*p* value	Cramer's *V*
*WORK *[%]						
Normotensives	30.4	44.2	5.7	*χ* ^2^ = 23.9	<.001	.402
Hypertensives	69.6	55.8	94.3			
*LEISURE *[%]						
Normotensives	45.9	65.3	11.3	*χ* ^2^ = 39.9	<.001	.519
Hypertensives	54.1	34.7	88.7			
*AWAKE *[%]						
Normotensives	35.1	51.6	5.7	*χ* ^2^ = 31.5	<.001	.461
Hypertensives	64.9	48.4	94.3			
*SLEEP *[%]						
Normotensives	76.4	91.6	49.1	*χ* ^2^ = 34.1	<.001	.480
Hypertensives	23.6	8.4	50.9			
*24-hour TOTAL *[%]						
Normotensives	37.8	54.7	7.5	*χ* ^2^ = 32.2	<.001	.500
Hypertensives	62.2	45.3	92.5			

*Note*. *n*: sample; [%]: frequencies in %; *χ*^2^: test statistic; significance threshold (two-tailed): *p* < .001, *p* < .01, and *p* < .05; Cramer's *V* (correlations 0.1–0.3: weak, 0.4–0.5: medium, and >0.5: strong).

**Table 3:** Predictors of hypertension by self-monitored home blood pressure measurement (home BPM).

Included variables	Unstandardised/regression coefficient B	Standardised error	Wald	df	*p* Value	Standardised/effect coefficient exp⁡(*B*)	95% confidence intervals exp⁡(*B*)	
							Lower bound	Upper bound
Sex	1.28	.48	7.24	1	.007	3.613	.11	.71
Body mass index (BMI)	.52	.46	1.30	1	.253	1.690	.69	4.16
Diagnoses								
WORK	1.15	.72	2.51	1	.113	3.152	.76	13.03
LEISURE	1.83	.56	10.77	1	.001	6.227	2.09	18.56
SLEEP	1.67	.55	9.21	1	.002	5.320	1.81	15.65
Constant	−2.79	.76	13.55	1	.001	.061		

*Note*.  Binary logistic regression (method: inclusion); dependent variable: hypertension diagnosis by home BPM; reference category = 0; sex: 0 = female; 1 = male, BMI classification: 0 = normal weight; 1 = overweight + obesity; sportive activity: 0 = regularly; 1= not at all + occasionally; 24-hr ABPM: diagnoses: 0 = normotension; 1 = hypertension during phases WORK-LEISURE-SLEEP; significance threshold (two-tailed): *p* < .001, *p* < .01, and *p* < .05; goodness-of-fit model's quality: 52%.
